# Cardiovascular Disease Burden and Outcomes Among American Indian and Alaska Native Medicare Beneficiaries

**DOI:** 10.1001/jamanetworkopen.2023.34923

**Published:** 2023-09-22

**Authors:** Lauren A. Eberly, Kaitlyn Shultz, Maricruz Merino, Maria Ynes Brueckner, Ernest Benally, Ada Tennison, Sabor Biggs, Lakotah Hardie, Ye Tian, Ashwin S. Nathan, Sameed Ahmed M. Khatana, Judy A. Shea, Eldrin Lewis, Gene Bukhman, Sonya Shin, Peter W. Groeneveld

**Affiliations:** 1 Gallup Indian Medical Center, Indian Health Service, Gallup, New Mexico; 2 Division of Cardiovascular Medicine, Department of Medicine, Hospital of the University of Pennsylvania, Philadelphia; 3 Penn Cardiovascular Outcomes, Quality, and Evaluative Research Center, Cardiovascular Institute, University of Pennsylvania, Philadelphia; 4 Penn Cardiovascular Center for Health Equity and Social Justice, University of Pennsylvania, Philadelphia; 5 Leonard Davis Institute of Health Economics, University of Pennsylvania, Philadelphia; 6 Division of General Internal Medicine, Massachusetts General Hospital, Boston; 7 Division of Pulmonary and Critical Care, Penn Presbyterian Medical Center, Philadelphia, Pennsylvania; 8 Division of General Internal Medicine, University of Pennsylvania, Philadelphia; 9 Division of Cardiovascular Medicine, Stanford University Medical Center, Palo Alto, California; 10 Division of Global Health Equity, Brigham and Women’s Hospital, Boston, Massachusetts; 11 Department of Global Health and Social Medicine, Program in Global Noncommunicable Diseases and Social Change, Harvard Medical School, Boston, Massachusetts; 12 Division of Cardiovascular Medicine, Brigham and Women’s Hospital, Boston, Massachusetts; 13 Corporal Michael J. Crescenz Veterans Affairs Medical Center, Philadelphia, Pennsylvania; 14 Division of General Internal Medicine, Perelman School of Medicine at the University of Pennsylvania, Philadelphia

## Abstract

**Importance:**

American Indian and Alaska Native persons face significant health disparities; however, data regarding the burden of cardiovascular disease in the current era is limited.

**Objective:**

To determine the incidence and prevalence of cardiovascular disease, the burden of comorbid conditions, including cardiovascular disease risk factors, and associated mortality among American Indian and Alaska Native patients with Medicare insurance.

**Design, Setting, and Participants:**

This was a population-based cohort study conducted from January 2015 to December 2019 using Medicare administrative data. Participants included American Indian and Alaska Native Medicare beneficiaries 65 years and older enrolled in both Medicare part A and B fee-for-service Medicare. Statistical analyses were performed from November 2022 to April 2023.

**Main Outcomes and Measures:**

The annual incidence, prevalence, and mortality associated with coronary artery disease (CAD), heart failure (HF), atrial fibrillation/flutter (AF), and cerebrovascular disease (stroke or transient ischemic attack [TIA]).

**Results:**

Among 220 598 American Indian and Alaska Native Medicare beneficiaries, the median (IQR) age was 72.5 (68.5-79.0) years, 127 402 were female (57.8%), 78 438 (38.8%) came from communities in the most economically distressed quintile in the Distressed Communities Index. In the cohort, 44.8% of patients (98 833) were diagnosed with diabetes, 61.3% (135 124) were diagnosed with hyperlipidemia, and 72.2% (159 365) were diagnosed with hypertension during the study period. The prevalence of CAD was 38.6% (61 125 patients) in 2015 and 36.7% (68 130 patients) in 2019 (*P* < .001). The incidence of acute myocardial infarction increased from 6.9 per 1000 person-years in 2015 to 7.7 per 1000 patient-years in 2019 (percentage change, 4.79%; *P* < .001). The prevalence of HF was 22.9% (36 288 patients) in 2015 and 21.4% (39 857 patients) in 2019 (*P* < .001). The incidence of HF increased from 26.1 per 1000 person-years in 2015 to 27.0 per 1000 person-years in 2019 (percentage change, 4.08%; *P* < .001). AF had a stable prevalence of 9% during the study period (2015: 9.4% [14 899 patients] vs 2019: 9.3% [25 175 patients]). The incidence of stroke or TIA decreased slightly throughout the study period (12.7 per 1000 person-years in 2015 and 12.1 per 1000 person-years in 2019; percentage change, 5.08; *P* = .004). Fifty percent of patients (110 244) had at least 1 severe cardiovascular condition (CAD, HF, AF, or cerebrovascular disease), and the overall mortality rate for the cohort was 19.8% (43 589 patients).

**Conclusions and Relevance:**

In this large cohort study of American Indian and Alaska Native patients with Medicare insurance in the US, results suggest a significant burden of cardiovascular disease and cardiometabolic risk factors. These results highlight the critical need for future efforts to prioritize the cardiovascular health of this population.

## Key Points

**Question:**

What are the incidence, prevalence, trends in disease burden, as well as associated mortality and comorbid conditions of cardiovascular disease among American Indian and Alaska Native Medicare beneficiaries between January 1, 2015, and December 30, 2019?

**Findings:**

In this cohort of 220 598 American Indian and Alaska Native Medicare beneficiaries, there was a high prevalence of cardiovascular disease and cardiovascular risk factors with nearly 50% of patients diagnosed with cardiovascular disease. From 2015 to 2019, coronary artery disease prevalence decreased, acute myocardial infarction and heart failure incidence increased, and the burden of cerebrovascular disease and atrial fibrillation remained relatively stable. The overall mortality rate was nearly 20% for the entire cohort.

**Meaning:**

In this study, the US American Indian and Alaska Native Medicare population had a high prevalence of cardiovascular disease and cardiovascular risk factors, suggesting the urgent need for implementation of strategies to advance cardiovascular equity for this population.

## Teaser

This cohort study investigates the incidence, prevalence, trends in disease burden, and associated mortality and comorbid conditions of cardiovascular disease among American Indian and Alaska Native Medicare beneficiaries from January 1, 2015, to December 30, 2019.

## Introduction

The American Indian and Alaska Native population has experienced significant health disparities compared with other racial and ethnic groups in the US.^1^ Pervasive structural racism, broken treaty obligations, settler colonialism, genocide, and exclusionary governmental policies have concentrated poverty and fueled health inequities among Indigenous populations in the US.^2,3,4,5^ Given this, American Indian and Alaska Native persons have a disproportionate burden of chronic disease, with the lowest life expectancy of all racial groups in the US.^6^ American Indian patients have been shown to have double the incidence of coronary heart disease compared with other racial groups.^7^ However, these epidemiologic data are over 2 decades old, and data regarding the burden and outcomes of other cardiovascular disease entities and their outcomes among the American Indian and Alaska Native population are limited. In fact, to our knowledge, there are no large cohort studies in the current era investigating the burden and outcomes of cardiovascular disease among American Indian and Alaska Native patients.

Improvements in health care in the US have not been equitably distributed, and there is evidence of worsening disparities in the quality of care and health outcomes among the American Indian and Alaska Native population.^8,9^ Furthermore, racial disparities in the major risk factors for cardiovascular disease, such as diabetes and hypertension, are widening.^10^ Accurate estimates of cardiovascular disease burden in this population and outcomes are critical to increase awareness of the cardiovascular health status of this population and inform future equity efforts. Therefore, in this study, we examined trends in the incidence and prevalence of cardiovascular disease including coronary artery disease (CAD), heart failure (HF), atrial fibrillation/flutter (AF), cerebrovascular disease, the burden of comorbid conditions including cardiovascular disease risk factors, as well as associated mortality among American Indian and Alaska Native Medicare beneficiaries.

## Methods

The University of Pennsylvania institutional review board approved this study and determined that this research was exempt from the regulatory requirements. Patient informed consent was not required owing to the use of deidentified patient data. We followed the Strengthening the Reporting of Observational Studies in Epidemiology (STROBE) reporting guidelines.

### Data Sources and Study Population

We used Medicare administrative data from the US Centers for Medicare & Medicaid Services (CMS), including enrollment and demographic data from the Medicare Beneficiary Summary File, claims data, and data from the Chronic Conditions Data Warehouse (CCW) for all American Indian and Alaska Native Medicare beneficiaries from 2015 through 2019. The Medicare Beneficiary Summary Files contain annually updated information regarding beneficiaries’ date of birth, race and ethnicity (from the Medicare enrollment database based on Social Security Administration records), sex, zip code of residence, date of death, monthly indicators of program eligibility and enrollment, including dual eligibility for Medicaid, and monthly indicators of enrollment in Medicare Managed Care. Patient zip code of residence was linked to Distressed Communities Index, a validated marker of socioeconomic status.^11^ The Distressed Communities Index combines 7 economic indicators (percentage of population with a high school diploma, housing vacancy rate, percentage of adults not working, poverty rate, median income ratio, change in employment, and change in business establishments) to generate a single index score, with a range from 0 (least distressed) to 100 (most distressed). We limited the analysis to beneficiaries 66 years or older living in the US, rather than the eligibility cuff of 65 years, to allow for a 1-year lookback period to assess comorbidities. Because claims data available in national databases are limited for Medicare Managed Care enrollees, we only included beneficiaries who were enrolled in fee-for-service Medicare. We only included beneficiaries who were enrolled in both Medicare Part A and Part B to ensure the most complete set of claims were available for each patient.

### Baseline Characteristics and Comorbidities

For all patients, we summarized baseline characteristics including comorbid conditions. The prevalence of comorbid conditions was determined based on if a patient met diagnostic criteria for each condition in the annual CCW. The CCW, developed by CMS, is derived from a comprehensive search of each beneficiary’s diagnosis and procedures codes report on both inpatient and outpatient claims during the calendar year, and it indicates whether a beneficiary had been diagnosed with 1 of 30 chronic conditions based on validated algorithms.^12,13^ Once a patient is diagnosed with a condition in a specific year in the CCW, they are assigned this diagnosis perpetually in each subsequent year until death.

### Incidence

We identified beneficiaries with cardiovascular disease, including CAD (defined as diagnosis of ischemic heart disease and/or acute myocardial infarction), HF, and AF based on if they met diagnostic criteria in the CCW. Diagnostic criteria for each condition in the CCW are summarized in eTable in Supplement 1. Diagnosis date for each condition is the earliest date that a patient met diagnostic criteria for the condition.^12^ We considered a beneficiary to have incident diagnosis for one of the cardiovascular disease conditions only if the diagnosis occurred after at least 1 year of enrollment in fee-for-service Medicare with no diagnosis in the 1-year lookback period and if the diagnosis did not occur in any of preceding years of the study period. We calculated annual incidence as the number of beneficiaries with incident diagnosis of each cardiovascular diagnosis during the year divided by the total person-years at risk among all beneficiaries in that year who did not previously have the diagnosis. Only person-time from beneficiaries with at least 1 year of enrollment in fee-for-service Medicare were included in the denominator. Therefore, to ensure that all patients had at least 1 year of prior claims history in Medicare, all incidence calculations reflect a population of 66 years or older. Patients who died were included in the annual incidence calculations for the year of death, but patients who changed insurance or dropped out of the cohort for reasons other than death during the year were excluded from incidence calculations for that specific year.

### Prevalence

We calculated annual prevalence on December 31 of each year as the number of beneficiaries alive with diagnosis divided by the total number of beneficiaries alive during that year. We determined that a beneficiary had a prevalent cardiovascular condition in a given year if the diagnosis was coded as previously described during that year. Similar to incidence calculations, only beneficiaries enrolled in fee-for-service Medicare for the entire calendar year were included in the prevalence calculations; thus, we would have an opportunity to observe claims with the diagnosis. To ensure that all patients had at least 1 year of prior claims history in Medicare, all prevalence calculations reflect a population of 66 years or older. Patients who died in a specific calendar year were included in the annual prevalence calculations for the year of death. Patients who changed insurance or dropped out of the cohort for reasons other than death during the calendar year were excluded from prevalence calculations for that specific year.

### Mortality

Death was determined from death dates in the Medicare Beneficiary Summary File; these are derived from the Social Security Death Master File. All patients alive and present in the cohort on January 1 of a given year were included in that year’s mortality calculations.

### Statistical Analysis

Summary statistics for patient characteristics are presented as medians with IQRs or means with SDs for continuous data and as total numbers and percentages for categorical data. To visualize geographic variation in the number of patients with cardiovascular disease (CAD, HF, AF, and/or cerebrovascular disease) and cardiovascular disease risk factors (hypertension, hyperlipidemia, and/or diabetes) among those with no cardiovascular disease diagnosis, we plotted choropleths, demonstrating the number of patients with cardiovascular disease and risk factors by zip code. We calculated 5-year and annual incidence and prevalence of each cardiovascular condition over the study period. We determined annual percentage change in incidence and prevalence rates and used negative binomial regression to evaluate the statistical significance of changes in incidence and prevalence rates over the 5-year study period.

We determined the mortality rate for each cardiovascular disease and annual mortality rates for the entire cohort. The disease specific annual mortality rate was determined by patients who died with the condition in 1 calendar year divided by patients that had the condition in that same calendar year. We performed negative binomial regression to test for temporal trends in mortality across the study period. We then similarly evaluated mortality in each subset separately: (1) CAD (ischemic heart disease and/or acute myocardial infarction), (2) HF, and (3) AF. All *P* values were 2-sided, and the cutoff for statistical significance was *P* <.05.

Statistical analyses were performed from November 2022 to April 2023 using SAS, version 9.4 (SAS Institute). Choropleths were generated using R Studio, version 1.3.959 (R Foundation for Statistical Computing).

## Results

### Baseline Characteristics

A total of 220 598 American Indian and Alaska Native Medicare beneficiaries met inclusion criteria. Baseline demographic and clinical characteristics of the cohort are summarized in Table 1. Of the 220 598 patients, 127 402 were female (57.8%), 93 196 (42.2%) were male, and the median (IQR) age was 72.5 (68.5-79.0) years. A total of 72 952 patients (33.1%) were dually eligible for both Medicare and Medicaid. For the overall cohort, the median (IQR) household income was $49 375 ($39 750-$62 648). The mean (SD) Distressed Communities Index was 64.0 (27.9), with 78 438 patients (38.8%) being in the highest, or most economically distressed, quintile.

**Table 1.** Baseline Characteristics of American Indian and Alaska Native Medicare Beneficiaries

Characteristic	Overall cohort (n = 220 598)
Age, median (IQR), y	72.5 (68.5-79.0)
Male, No. (%)	93 196 (42.2)
Female, No. (%)	127 402 (57.8)
Dual eligibility with Medicaid, No. (%)	72 952 (33.1)
Distressed Communities Index, mean (SD)^a^	64.0 (27.9)
Distressed Communities Index quintile (5 = most economically distressed), No. (%)	
1	20 765 (10.3)
2	23 874 (11.8)
3	31 759 (15.7)
4	47 343 (23.4)
5	78 438 (38.8)
Region of residence, No. (%)	
Midwest	29 817 (14.8)
Northeast	8202 (4.1)
South	65 735 (32.5)
West	98 425 (48.7)
Household income, median (IQR), $^b^	49 375 (39 750-62 648)
Comorbidities, No. (%)	
Diabetes	98 833 (44.8)
Hyperlipidemia	135 124 (61.3)
Hypertension	159 365 (72.2)
Chronic kidney disease	85 459 (38.7)

^a^ Missing data: Distressed Community Index: 18 419 (8.4%).

^b^ Missing data: median household income: 7348 (3.3%).

There were high rates of chronic conditions and cardiovascular risk factors at baseline. Among all members of the cohort, 98 833 patients (44.8%) had a diagnosis of diabetes, 135 124 (61.3%) had a diagnosis of hyperlipidemia, and 159 365 (72.2%) had a diagnosis of hypertension during the 5-year study period. A total of 85 459 patients (38.7%) had a diagnosis of chronic kidney disease. Choropleths with the number of patients with cardiovascular disease (HF, CAD, AF, and/or cerebrovascular disease) and cardiovascular risk factors (hypertension, hyperlipidemia, and/or diabetes) among those with no cardiovascular disease diagnosis by zip code nationally are shown in the Figure. The rate of cardiovascular disease conditions and cardiovascular risk factors by sex are shown in eFigure 1 in Supplement 1. For those without cardiovascular disease (CAD, CHF, AF, or cerebrovascular disease), the rate of cardiovascular disease risk factors by sex are shown in eFigure 2 in Supplement 1.

**Figure.**
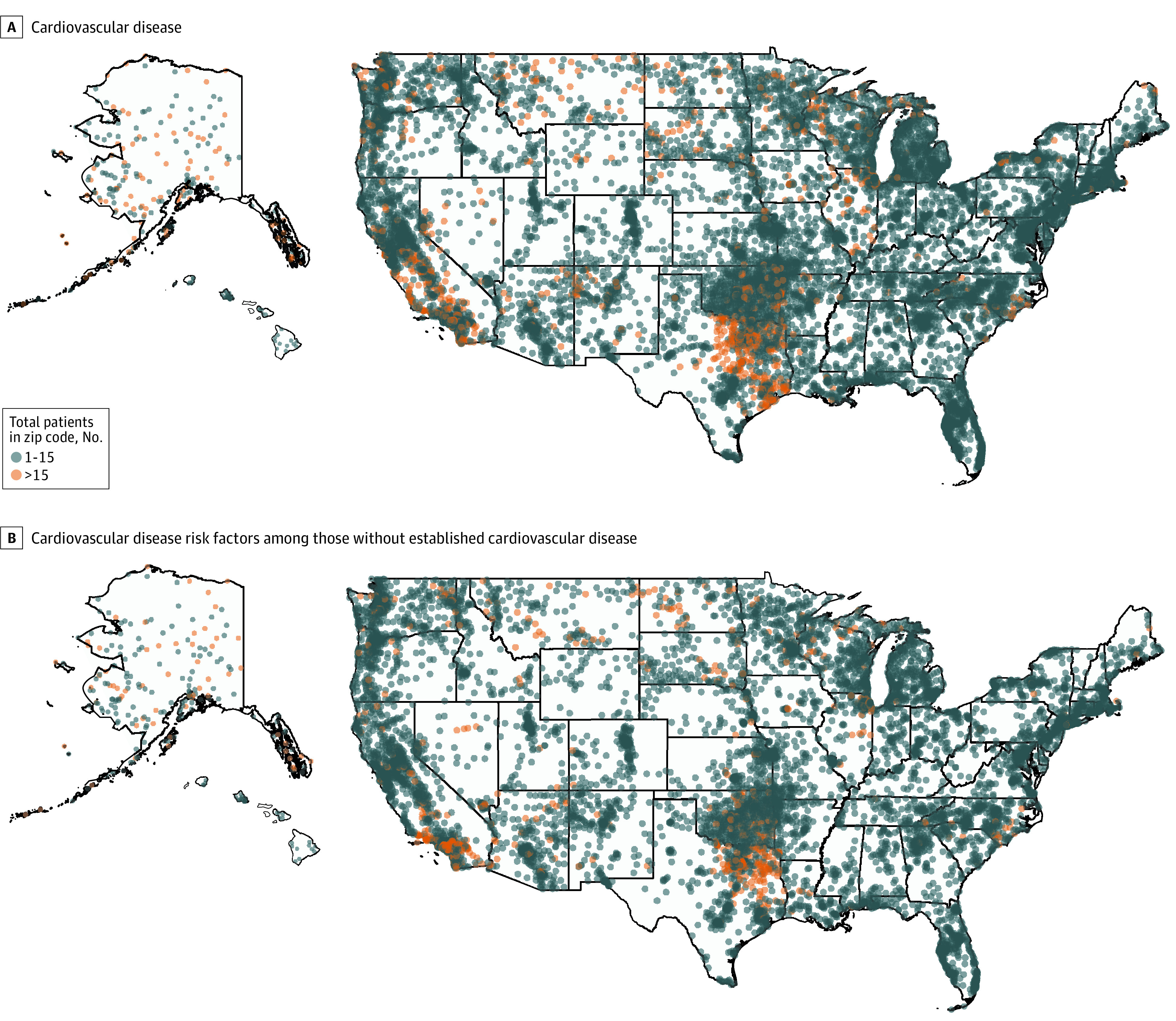
Geographic Variation in Cardiovascular Disease and Cardiovascular Risk Factors Among American Indian and Alaska Native Medicare Beneficiaries Nationally Choropleths demonstrate geographic variation in the number of patients with cardiovascular disease (A) and those with cardiovascular risk factors without established cardiovascular disease (B) by zip code nationally among American Indian and Alaska Native Medicare beneficiaries.

### Prevalence

Prevalence rates by year and overall for the study period are summarized in Table 2. Over the study period, 110 244 patients (49.9%) had at least 1 severe cardiovascular condition (CAD, HF, AF, or cerebrovascular disease). The prevalence of CAD was 38.6% (61 125 patients) in 2015 and 36.7% (68 130 patients) in 2019 (*P* < .001). The prevalence of HF decreased slightly during study the period with a prevalence of 22.9% (36 288 patients) in 2015 and 21.1% (39 857 patients) in 2019 (*P* < .001). The prevalence of AF was relatively stable with a rate of 9.4% (14 899 patients) in 2015 and 9.3% (25 175 patients) in 2019 (*P* < .001). The prevalence of cerebrovascular disease slightly decreased throughout the study period (11.7% [18 613 patients] in 2015 and 11.0% [20 522 patients] in 2019; *P* < .001).

**Table 2.** Annual Prevalence of Cardiovascular Disease Subtypes and Change Over Time for the Entire Cohort, 2015-2019

Disease	2015	2016	2017	2018	2019	Overall	Change, %	*P* value
Atrial fibrillation or flutter, No. (%)	14 899 (9.4)	15 535 (9.39)	16 251 (9.36)	16 826 (9.34)	17 316 (9.32)	25 175 (11.41)	3.86	<.001
HF, No. (%)	36 288 (22.89)	37 137 (22.44)	38 121 (21.97)	38 931 (21.62)	39 857 (21.44)	59 439 (26.94)	2.37	<.001
Cerebrovascular disease, No. (%)	18 613 (11.74)	19 190 (11.6)	19 707 (11.36)	20 118 (11.17)	20 522 (11.04)	30 231 (13.70)	2.45	<.001
CAD, No. (%)	61 125 (38.55)	62 784 (37.94)	64 782 (37.33)	66 305 (36.82)	68 130 (36.65)	90 639 (41.09)	2.75	<.001
Any cardiovascular condition (atrial fibrillation/flutter, HF, cerebrovascular disease, CAD), No. (%)	74 306 (46.86)	76 397 (46.16)	78 994 (45.52)	80 963 (44.96)	83 140 (44.73)	110 244 (49.98)	2.86	<.001

Abbreviations: CAD, coronary artery disease; HF, heart failure.

### Incidence

Incidence rates of cardiovascular disease subtypes by year for the study period are summarized in Table 3. The incidence of HF increased from 26.1 per 1000 person-years in 2015 to 27.0 per 1000 person-years in 2019 (percentage change, 4.08%; *P* < .001). The incidence of CAD increased from 37.2 per 1000 person-years in 2015 to 41.5 per 1000 person-years in 2017 and then decreased to 35.3 per 1000 person-years in 2019 (*P* < .001). The incidence of acute myocardial infarction increased from 6.9 to 7.7 per 1000 person-years from 2015 to 2019 (percentage change, 4.79%; *P* = .001). The incidence of AF slightly decreased throughout the study period with an incidence of 10.98 per 1000 person-years in 2015 and 10.55 per 1000 person-years in 2019 (percentage change, 2.43%; *P* < .001). The incidence of stroke/transient ischemic attack slightly decreased during the study period at 12.7 per 1000 person-years in 2015 and 12.1 per 1000 person-years in 2019 (percentage change, 5.08; *P* = .004).

**Table 3.** Annual Incidence of Cardiovascular Disease Subtypes (Incidence Rate per 1000 Person-Years) and Change Over Time for the Entire Cohort, 2015-2019

Disease	Incidence rate per 1000 person-years					Change, %	*P* value
	2015	2016	2017	2018	2019		
Atrial fibrillation and flutter	10.98	11.39	11.62	11.23	10.55	2.43	.001
HF	26.09	28.02	29.12	28.09	27.01	4.08	<.001
Stroke or TIA	12.69	12.74	12.82	12.43	12.13	5.08	.004
CAD	37.16	39.22	41.5	36.49	35.28	5.08	<.001
Acute myocardial infarction	6.9	7.3	7.8	7.7	7.7	4.79	<.001

Abbreviations: CAD, coronary artery disease; HF, heart failure; TIA, transient ischemic attack.

### Mortality

There were 43 589 deaths during the study period, with an overall mortality rate of 19.8% (43 589 patients). For those who entered the cohort in 2015, the 5-year mortality rate was 25.5%. All-cause mortality by year for the entire cohort and all-cause mortality for patients with CAD, HF, AF, and cerebrovascular disease are summarized in Table 4.

**Table 4.** All-Cause Mortality Rate Overall and by Cardiovascular Disease Subtype, 2015-2019

Disease	2015	2016	2017	2018	2019	Overall	Change, %	*P* value
Overall cohort, No. (%)	8214 (5.18)	8356 (5.05)	8778 (5.06)	8984 (4.99)	9257 (4.98)	43 589 (19.76)	3.16	<.001
CAD, No. (%)	5336 (8.73)	5521 (8.79)	5215 (8.05)	5825 (8.79)	5914 (8.68)	28 283 (31.2)	2.63	.009
HF, No. (%)	4682 (12.90)	4785 (12.88)	4969 (13.03)	5035 (12.93)	5067 (12.71)	24 538 (41.28)	2.10	<.001
Cerebrovascular disease, No. (%)	2289 (12.3)	2397 (12.49)	2458 (12.47)	2496 (12.41)	2500 (12.18)	12 140 (40.16)	2.17	.001
Atrial fibrillation or flutter, No. (%)	1848 (12.4)	1899 (12.22)	2010 (12.37)	2065 (12.27)	2120 (12.24)	9942 (39.49)	3.64	<.001
Cardiovascular disease, No. (%)^a^	6470 (8.71)	6593 (8.63)	6867 (8.69)	6992 (8.64)	7129 (8.57)	34 051 (30.89)	2.52	<.001

Abbreviations: CAD, coronary artery disease; HF, heart failure.

^a^ Had diagnosis of CAD, HF, cerebrovascular disease, and/or atrial fibrillation/flutter.

## Discussion

In this large cohort study of American Indian and Alaska Native patients with Medicare insurance in the US, results suggest a significant burden of cardiovascular disease and cardiometabolic risk factors. Nearly 50% of the patients had a diagnosis of a severe cardiovascular condition. The prevalence of CAD and HF were high, with over 40% of patients having a diagnosis of CAD and 27% of patients having a diagnosis of HF during the study period. Incidence of HF and acute myocardial infarction increased during the study period. Mortality rates were high overall and for patients with cardiovascular disease. The results of this study provide needed epidemiologic insight into the cardiovascular disease burden among the American Indian and Alaska Native Medicare population in the US and highlight the critical need for future equity efforts to prioritize the cardiovascular health of this population.

The large burden of cardiovascular and cerebrovascular disease in this cohort is consistent with the Strong Heart Study, previously the largest population-based cohort study of the American Indian and Alaska Native population. The Strong Heart Study, which included a geographically diverse group of American Indian patients, demonstrated a high burden of coronary artery disease, with double the incidence of other racial groups, as well as a higher incidence of stroke compared with the US Black and White population at that time.^7,14^ Our results come 2 decades after the Strong Heart Study results and suggest a persistently high burden of ischemic heart and cerebrovascular disease. Other forms of cardiovascular disease, such as AF and HF, have previously been severely understudied in this population.^5^ Our results indicate that American Indian and Alaska Native patients may be disproportionately affected by these conditions as well.^15,16^

These results may reflect the marked and pervasive racial inequities in cardiovascular health in the US.^8,9,15,16^ Although we did not directly compare incidence and prevalence of these conditions to other racial groups in this study limiting definitive conclusions, previously published data have demonstrated a prevalence of ischemic heart disease for all Medicare fee-for-service beneficiaries of 26.8% in 2019, as compared with 37% in our cohort.^17^ Similarly, the HF prevalence rate has been shown to be 14.5% among all Medicare fee-for-service beneficiaries and 14% among White patients in 2018,^18^ as compared with 22% for the same year in our population, raising concern of a disparate burden of this highly morbid cardiovascular condition among this population. Additionally, the demonstrated rising incidence of HF in the study population is alarming and is notably in clear contrast to prior evidence of a declining incidence of HF among all Medicare beneficiaries.^19^

The high burden of cardiovascular disease in our cohort is not surprising given the high prevalence of risk factors for cardiovascular disease development in the cohort, including hypertension, diabetes, and hyperlipidemia. In fact, nearly one-half of patients had diabetes in the cohort. The high rate of hypertension and diabetes is consistent with prior National Health Interview Survey and Behavioral Risk Factor Surveillance System survey data evaluating rates of self-reported diagnosis in the population,^20,21,22^ as well as data among the Indian Health Service clinical population.^23^ Although data regarding hyperlipidemia in the American Indian and Alaska Native population have been mixed in prior studies,^24,25,26^ our results suggest a high prevalence of this condition among older adults. Diabetes has been demonstrated to be the most important risk factor for development of ischemic heart disease among American Indian patients.^7^ Additionally, diabetes has been shown to independently impact cardiac structure and function in American Indian populations, including leading to increased left ventricular (LV) wall thickness, reduced LV systolic function, and increased arterial stiffness, all which contribute to the development of HF and arrhythmias.^27,28^ Hypertension, similarly, is a strong independent risk factor for cardiovascular disease and LV systolic dysfunction in American Indian and Alaska Native populations.^29^ Given that racial disparities in the major risk factors for cardiovascular disease, such as diabetes and hypertension, are widening in the US, inequities in the burden of cardiovascular disease will continue to worsen over time without immediate implementation of preventative strategies that prioritize the health of racially marginalized patient groups.^30^

It is important to acknowledge the sociopolitical, environmental, and economic inequities, rooted in racism, that drive the study findings.^31,32,33,34,35^ The burden of cardiovascular disease must be contextualized within a long racist history of settler colonialism, Indigenous genocide, governmental relocation policies, broken treaty obligations, intergenerational trauma, and intentional and sustained community disinvestment, all of which have fueled poverty and poor health in this population.^2,3,4,36,37^ This is reflected in nearly 40% of the patients in our cohort falling into the most economically distressed category based on the Distressed Communities Index. Entrenched poverty drives poor health.^38,39^ Traditionally, Indigenous people lived a healthy lifestyle with high rates of physical activity and consumption of traditional, healthy foods. However, as a result of harmful and racist governmental policies and practices over the last several centuries, food insecurity is endemic in many Indigenous communities^40,41,42^—of the 27 000 square miles of land on the Navajo Nation, the largest US reservation, there are only 13 grocery stores.^43^ Food insecurity leads to high rates of obesity, dyslipidemia, and diabetes and is independently associated with cardiovascular health.^44^ We are long overdue for a reckoning to address the historical and ongoing harm within tribal communities; efforts to rectify this long legacy of injustices are necessary to combat health inequities.^45,46,47^

Solutions to improve cardiovascular health must be community designed and community led and must center tribal sovereignty.^48^ Investing in community-level solutions to address the high burden of cardiovascular risk factors is of critical importance. Prescription programs of healthy fruits and vegetables and policies like the Navajo Nation Healthy Diné Nation Act or junk food tax are examples of effective community-based ways to address food sovereignty and increase access to nutritious foods.^49,50^ Genuine engagement of Indigenous communities, with strategies that integrate traditional cultural teaching and healing into Western medicine, are essential to achieve optimal health.^51^ Promotion of Indigenous knowledge and traditional cultural practices has been shown to reduce chronic diseases in some American Indian and Alaska Native communities.^52^ For example, strategies that incorporate tribal history, language, and craft making have been shown to be more effective for weight loss and glycemic control than standard physical activity recommendations and diet intervention.^53^ Expansion of multilevel interventions in health care that address individual- and community-level factors, such as the Special Diabetes Program for Indians, to improve cardiovascular health are needed.^54^ However, these strategies must be coupled with targeted investment in communities to address the effect of sociopolitical determinants on health.^31,46,55^

### Limitations

Our study has several limitations. It is critical to note that the race and ethnicity variable in the Medicare database has been shown to have a low validity for certain racial groups, particularly for those of American Indian and Alaska Native race.^56,57^ It is possible that a proportion of the included patients are misclassified and are not, in fact, American Indian or Alaska Native. Additionally, because only 1 racial category can be selected on enrollment, this may lead to underrepresentation or exclusion of multiracial patients. Further studies of this population with improved measures of self-identified race are needed to confirm these results. Our results only represent Medicare beneficiaries and may not be reflective of American Indian and Alaska Native patients with other payor types. Population estimates are based on administrative claims, and there may be errors in coding. If medical encounters are infrequent and/or coding incomplete, diagnoses may be missed. However, the current Medicare algorithms for diagnosis of chronic conditions are well validated.^13^ Incidence and prevalence estimates do not reflect the true population-based burden. However, they do reflect the burden of these conditions within Medicare. Considering that American Indians and Alaska Natives have been shown to have worse access to care for 62% of access measures,^1^ these results likely underestimate the true burden of disease. This study was limited to Medicare claims, and therefore, beneficiaries who were diagnosed with a condition over 1 year before enrollment may have been misclassified. The American Indian and Alaska Native population represent a heterogeneous and diverse group. We could not evaluate differences based on tribal affiliations, which have been shown to be associated with cardiovascular disease risk.^7^

## Conclusions

In the largest, to our knowledge, population-based epidemiologic cohort study of the American Indians and Alaska Native population to date, results suggest a high burden of cardiovascular disease and risk factors among Medicare beneficiaries. The path forward requires engagement to support community-lead initiatives and targeted investment in Indigenous communities to rectify historical harms and address sociopolitical determinants of cardiovascular health.

## Notes

Supplement 1. **eTable.** Diagnostic Criteria for Chronic Conditions Based on National CMS Medicare and Medicaid Algorithms **eFigure 1.** Rates of Overall Cardiovascular Disease by Gender and Rates of Overall Cardiovascular Disease Risk Factors by Gender for the Entire Cohort, 2015-2019 **eFigure 2.** Rates of Overall Cardiovascular Disease Risk Factors by Gender for Patients Without Established Cardiovascular Disease, 2015-2019

Supplement 2.
Data Sharing Statement
