# Supplementary material for: Cardiovascular Disease Burden and Outcomes Among American Indian and Alaska Native Medicare Beneficiaries
Source: JAMA Netw Open. 2023 Sep 22;6(9):e2334923. doi: 10.1001/jamanetworkopen.2023.34923 (PMC10517375; doi:10.1001/jamanetworkopen.2023.34923)
Supplement: Supplement 1. — eTable. Diagnostic Criteria for Chronic Conditions Based on National CMS Medicare and Medicaid Algorithms eFigure 1. Rates of Overall Cardiovascular Disease by Gender and Rates of Overall Cardiovascular Disease Risk Factors by Gender for the Entire Cohort, 2015-2019 eFigure 2. Rates of Overall Cardiovascular Disease Risk Factors by Gender for Patients Without Established Cardiovascular Disease, 2015-2019 [file jamanetwopen-e2334923-s001.pdf]

Supplementary Online Content

Eberly LA, Shultz K, Merino M, et al. Cardiovascular disease burden and outcomes among American Indian and Alaska Native Medicare beneficiaries. *JAMA Netw Open*. 2023;6(9):e2334923. doi:10.1001/jamanetworkopen.2023.34923

eTable. Diagnostic Criteria for Chronic Conditions Based on National CMS Medicare and Medicaid Algorithms

eFigure 1. Rates of Overall Cardiovascular Disease by Gender and Rates of Overall Cardiovascular Disease Risk Factors by Gender for the Entire Cohort, 2015-2019

eFigure 2. Rates of Overall Cardiovascular Disease Risk Factors by Gender for Patients Without Established Cardiovascular Disease, 2015-2019

This supplementary material has been provided by the authors to give readers additional information about their work.

© 2023 Eberly LA et al. *JAMA Network Open.*

eTable. Diagnostic Criteria for Chronic Conditions Based on National CMS Medicare and Medicaid Algorithms

Condition	Reference Period	Number/Type of Claim to Qualify	Valid ICD-10 codes
Acute Myocardial Infarction	1 year	At least 1 inpatient claim with DX codes	I23.0, I23.1, I23.2, I23.3, I23.4, I23.5, I23.6, I23.7, I23.8, I21.01, I21.02, I21.09, I21.11, I21.19, I21.21, I21.29, I21.3, I21.4, I21.9, I21.A1, I21.A9, I22.0, I22.1, I22.2, I22.8, I22.9 (any DX on the claim)
Atrial fibrillation and Flutter	2 years	At least 1 inpatient/SNF/HHA claim OR 2 HOP/carrier claims with DX codes	I48.0, I48.1, I48.11, I48.19, I48.2, I48.20, I48.21, I48.3, I48.4, I48.91 (any DX on the claim)
Heart Failure	2 years	At least 1 inpatient/SNF/HHA claim OR 2 HOP/carrier claims with DX codes	I09.81, I11.0, I13.0, I13.2, I42.0, I42.5, I42.6, I42.7, I42.8, I43, I50.1, I50.20, I50.21, I50.22, I50.23, I50.30, I50.31, I50.32, I50.33, I50.40, I50.41, I50.42, I50.43, I50.810, I50.811, I50.812, I50.813, I50.814, I50.82, I50.83, I50.84, I50.89, I50.9, P29.0 (any DX on the claim)
Ischemic Heart Disease	2 years	At least 1 inpatient/SNF/HHA claim OR 2 HOP/carrier claims with DX codes	I20.0, I20.1, I20.8, I24.0, I24.1, I24.8, I25.10, I25.110, I25.111, I25.118, I25.119, I25.3, I25.41, I25.42, I25.5, I25.6, I25.700, I25.701, I25.708, I25.710, I25.711, I25.718, I25.719, I25.720, I25.721, I25.728, I25.729, I25.730, I25.731, I25.738, I25.739, I25.750, I25.751, I25.758, I25.759, I25.760, I25.761, I25.768, I25.769, I25.790, I25.791, I25.798, I25.799, I25.810, I25.811, I25.812, I25.82, I25.83, I25.84, I25.89, I25.9 (any DX on the claim)
Cerebrovascular Disease (Stroke/Transient Ischemic Attack)	1 year	At least 1 inpatient, HOP or carrier claim with DX codes	G45.0, G45.1, G45.2, G45.3, G45.8, G45.9, G46.0, G46.1, G46.2, G46.3, G46.4, G46.5, G46.6, G46.7, G46.8, G97.31, G97.32, I60.00, I60.01, I60.02, I60.10, I60.11, I60.12, I60.2, I60.20, I60.21, I60.22, I60.30, I60.31, I60.32, I60.4, I60.50, I60.51, I60.52, I60.6, I60.7, I60.8, I60.9, I61.0, I61.1, I61.2, I61.3, I61.4, I61.5, I61.6, I61.8, I61.9, I62.00, I62.01, I62.02, I62.9, I63.00, I63.011, I63.012, I63.013, I63.019, I63.02, I63.031, I63.032, I63.033, I63.039, I63.09, I63.10, I63.111, I63.112, I63.113, I63.119, I63.12, I63.131, I63.132,

© 2023 Eberly LA et al. *JAMA Network Open.*

			I63.133, I63.139, I63.19, I63.20, I63.211, I63.212, I63.213, I63.219, I63.22, I63.231, I63.232, I63.233, I63.239, I63.29, I63.30, I63.311, I63.312, I63.313, I63.319, I63.321, I63.322, I63.323, I63.329, I63.331, I63.332, I63.333, I63.339, I63.341, I63.342, I63.343, I63.349, I63.39, I63.40, I63.411, I63.412, I63.413, I63.419, I63.421, I63.422, I63.423, I63.429, I63.431, I63.432, I63.433, I63.439, I63.441, I63.442, I63.443, I63.449, I63.49, I63.50, I63.511, I63.512, I63.513, I63.519, I63.521, I63.522, I63.523, I63.529, I63.531, I63.532, I63.533, I63.539, I63.541, I63.542, I63.543, I63.549, I63.59, I63.6, I63.8, I63.81, I63.89, I63.9, I67.841, I67.848, I67.89, I97.810, I97.811, I97.820, I97.821 (any DX on the claim)<br>EXCLUSION: If any of the qualifying claims have any of the following codes in any DX position then EXCLUDE: S06.340A, S06.341A, S06.342A, S06.343A, S06.344A, S06.345A, S06.346A, S06.347A, S06.348A, S06.349A, S06.350A, S06.351A, S06.352A, S06.353A, S06.354A, S06.355A, S06.356A, S06.357A, S06.358A, S06.359A, S06.360A, S06.361A, S06.362A, S06.363A, S06.364A, S06.365A, S06.366A, S06.367A, S06.368A, S06.369A, S06.370A, S06.371A, S06.372A, S06.373A, S06.374A, S06.375A, S06.376A, S06.377A, S06.378A, S06.379A, S06.380A, S06.381A, S06.382A, S06.383A, S06.384A, S06.385A, S06.386A, S06.387A, S06.388A, S06.389A, S06.5X0A, S06.5X1A, S06.5X2A, S06.5X3A, S06.5X4A, S06.5X5A, S06.5X6A, S06.5X7A, S06.5X8A, S06.5X9A, S06.6X0A, S06.6X1A, S06.6X2A, S06.6X3A, S06.6X4A, S06.6X5A, S06.6X6A, S06.6X7A, S06.6X8A, S06.6X9A, S06.810A, S06.811A, S06.812A, S06.813A, S06.814A, S06.815A, S06.816A, S06.817A,

© 2023 Eberly LA et al. *JAMA Network Open.*

			S06.818A, S06.819A, S06.820A, S06.821A, S06.822A, S06.823A, S06.824A, S06.825A, S06.826A, S06.827A, S06.828A, S06.829A, S06.890A, S06.891A, S06.892A, S06.893A, S06.894A, S06.895A, S06.896A, S06.897A, S06.898A, S06.899A, S06.9X0A, S06.9X1A, S06.9X2A, S06.9X3A, S06.9X4A, S06.9X5A, S06.9X6A, S06.9X7A, S06.9X8A, S06.9X9A, S06.A0XA, S06.A1XA
Diabetes	2 years	At least 1 inpatient/SNF/HHA claim OR 2 HOP/carrier claims with DX codes	E08.00, E08.01, E08.10, E08.11, E08.21, E08.22, E08.29, E08.311, E08.319, E08.321, E08.3211, E08.3212, E08.3213, E08.3219, E08.329, E08.3291, E08.3292, E08.3293, E08.3299, E08.331, E08.3311, E08.3312, E08.3313, E08.3319, E08.339, E08.3391, E08.3392, E08.3393, E08.3399, E08.341, E08.3411, E08.3412, E08.3413, E08.3419, E08.349, E08.3491, E08.3492, E08.3493, E08.3499, E08.351, E08.3511, E08.3512, E08.3513, E08.3519, E08.3521, E08.3522, E08.3523, E08.3529, E08.3531, E08.3532, E08.3533, E08.3539, E08.3541, E08.3542, E08.3543, E08.3549, E08.3551, E08.3552, E08.3553, E08.3559, E08.359, E08.3591, E08.3592, E08.3593, E08.3599, E08.36, E08.37X1, E08.37X2, E08.37X3, E08.37X9, E08.39, E08.40, E08.41, E08.42, E08.43, E08.44, E08.49, E08.51, E08.52, E08.59, E08.610, E08.618, E08.620, E08.621, E08.622, E08.628, E08.630, E08.638, E08.641, E08.649, E08.65, E08.69, E08.8, E08.9, E09.00, E09.01, E09.10, E09.11, E09.21, E09.22, E09.29, E09.311, E09.319, E09.321, E09.3211, E09.3212, E09.3213, E09.3219, E09.329, E09.3291, E09.3292, E09.3293, E09.3299, E09.331, E09.3311, E09.3312, E09.3313, E09.3319, E09.339, E09.3391, E09.3392, E09.3393, E09.3399, E09.341, E09.3411, E09.3412, E09.3413, E09.3419, E09.349, E09.3491, E09.3492, E09.3493, E09.3499, E09.351, E09.3511, E09.3512, E09.3513,

© 2023 Eberly LA et al. *JAMA Network Open.*

			E09.3519, E09.3521, E09.3522, E09.3523, E09.3529, E09.3531, E09.3532, E09.3533, E09.3539, E09.3541, E09.3542, E09.3543, E09.3549, E09.3551, E09.3552, E09.3553, E09.3559, E09.359, E09.3591, E09.3592, E09.3593, E09.3599, E09.36, E09.37X1, E09.37X2, E09.37X3, E09.37X9, E09.39, E09.40, E09.41, E09.42, E09.43, E09.44, E09.49, E09.51, E09.52, E09.59, E09.610, E09.618, E09.620, E09.621, E09.622, E09.628, E09.630, E09.638, E09.641, E09.649, E09.65, E09.69, E09.8, E09.9, E10.10, E10.11, E10.21, E10.22, E10.29, E10.311, E10.319, E10.321, E10.3211, E10.3212, E10.3213, E10.3219, E10.329, E10.3291, E10.3292, E10.3293, E10.3299, E10.331, E10.3311, E10.3312, E10.3313, E10.3319, E10.339, E10.3391, E10.3392, E10.3393, E10.3399, E10.341, E10.3411, E10.3412, E10.3413, E10.3419, E10.349, E10.3491, E10.3492, E10.3493, E10.3499, E10.351, E10.3511, E10.3512, E10.3513, E10.3519, E10.359, E10.36, E10.37X1, E10.37X2, E10.37X3, E10.37X9, E10.39, E10.40, E10.41, E10.42, E10.43, E10.44, E10.49, E10.51, E10.52, E10.59, E10.610, E10.618, E10.620, E10.621, E10.622, E10.628, E10.630, E10.638, E10.641, E10.649, E10.65, E10.69, E10.8, E10.9, E11.00, E11.01, E11.10, E11.11, E11.21, E11.22, E11.29, E11.311, E11.319, E11.321, E11.3211, E11.3212, E11.3213, E11.3219, E11.329, E11.3291, E11.3292, E11.3293, E11.3299, E11.331, E11.3311, E11.3312, E11.3313, E11.3319, E11.339, E11.3391, E11.3392, E11.3393, E11.3399, E11.341, E11.3411, E11.3412, E11.3413, E11.3419, E11.349, E11.3491, E11.3492, E11.3493, E11.3499, E11.351, E11.3511, E11.3512, E11.3513, E11.3519, E11.3521, E11.3522, E11.3523, E11.3529, E11.3531, E11.3532, E11.3533, E11.3539, E11.3541, E11.3542, E11.3543, E11.3549, E11.3551, E11.3552,

© 2023 Eberly LA et al. *JAMA Network Open.*

			E11.3553, E11.3559, E11.359, E11.3591, E11.3592, E11.3593, E11.3599, E11.36, E11.37X1, E11.37X2, E11.37X3, E11.37X9, E11.39, E11.40, E11.41, E11.42, E11.43, E11.44, E11.49, E11.51, E11.52, E11.59, E11.610, E11.618, E11.620, E11.621, E11.622, E11.628, E11.630, E11.638, E11.641, E11.649, E11.65, E11.69, E11.8, E11.9, E13.00, E13.01, E13.10, E13.11, E13.21, E13.22, E13.29, E13.311, E13.319, E13.321, E13.3211, E13.3212, E13.3213, E13.3219, E13.329, E13.3291, E13.3292, E13.3293, E13.3299, E13.331, E13.3311, E13.3312, E13.3313, E13.3319, E13.339, E13.3391, E13.3392, E13.3393, E13.3399, E13.341, E13.3411, E13.3412, E13.3413, E13.3419, E13.349, E13.3491, E13.3492, E13.3493, E13.3499, E13.351, E13.3511, E13.3512, E13.3513, E13.3519, E13.3521, E13.3522, E13.3523, E13.3529, E13.3531, E13.3532, E13.3533, E13.3539, E13.3541, E13.3542, E13.3543, E13.3549, E13.3551, E13.3552, E13.3553, E13.3559, E13.359, E13.36, E13.39, E13.40, E13.41, E13.42, E13.43, E13.44, E13.49, E13.51, E13.52, E13.59, E13.610, E13.618, E13.620, E13.621, E13.622, E13.628, E13.630, E13.638, E13.641, E13.649, E13.65, E13.69, E13.8, E13.9 (any DX on the claim)
Hyperlipidemia	2 years	At least 1 inpatient/SNF/HHA claim OR 2 HOP/carrier claims with DX codes	E78.0, E78.00, E78.01, E78.1, E78.2, E78.3, E78.4, E78.41, E78.49, E78.5 (any DX on the claim)
Hypertension	2 years	At least 1 inpatient/SNF/HHA claim OR 2 HOP/carrier claims with DX codes	H35.031, H35.032, H35.033, H35.039, I10, I11.0, I11.9, I12.0, I12.9, I13.0, I13.10, I13.11, I13.2, I15.0, I15.1, I15.2, I15.8, I15.9, I67.4, N26.2 (any DX on the claim)

SNF refers to skilled nursing facility; HHA refers to home health agency; HOP refers to hospital outpatient. Carrier claims refer to claim types 71 and 72 (not durable medical equipment [DME] claim types 81 or 82), and excludes any claims for which line item Berenson-Eggers Type of Service (BETOS) code variable equals D1A, D1B, D1C, D1D, D1E, D1F, D1G (which is DME), or O1A (which is ambulance services). The intent of the algorithm is to exclude claims where the services do not require a licensed health care professional. When two claims are required, they must occur at least one day apart.

© 2023 Eberly LA et al. *JAMA Network Open.*

eFigure 1. Rates of Overall Cardiovascular Disease by Gender and Rates of Overall Cardiovascular Disease Risk Factors by Gender for the Entire Cohort, 2015-2019

A. Rates of Overall Cardiovascular Disease by Gender for the Entire Cohort, 2015-2019

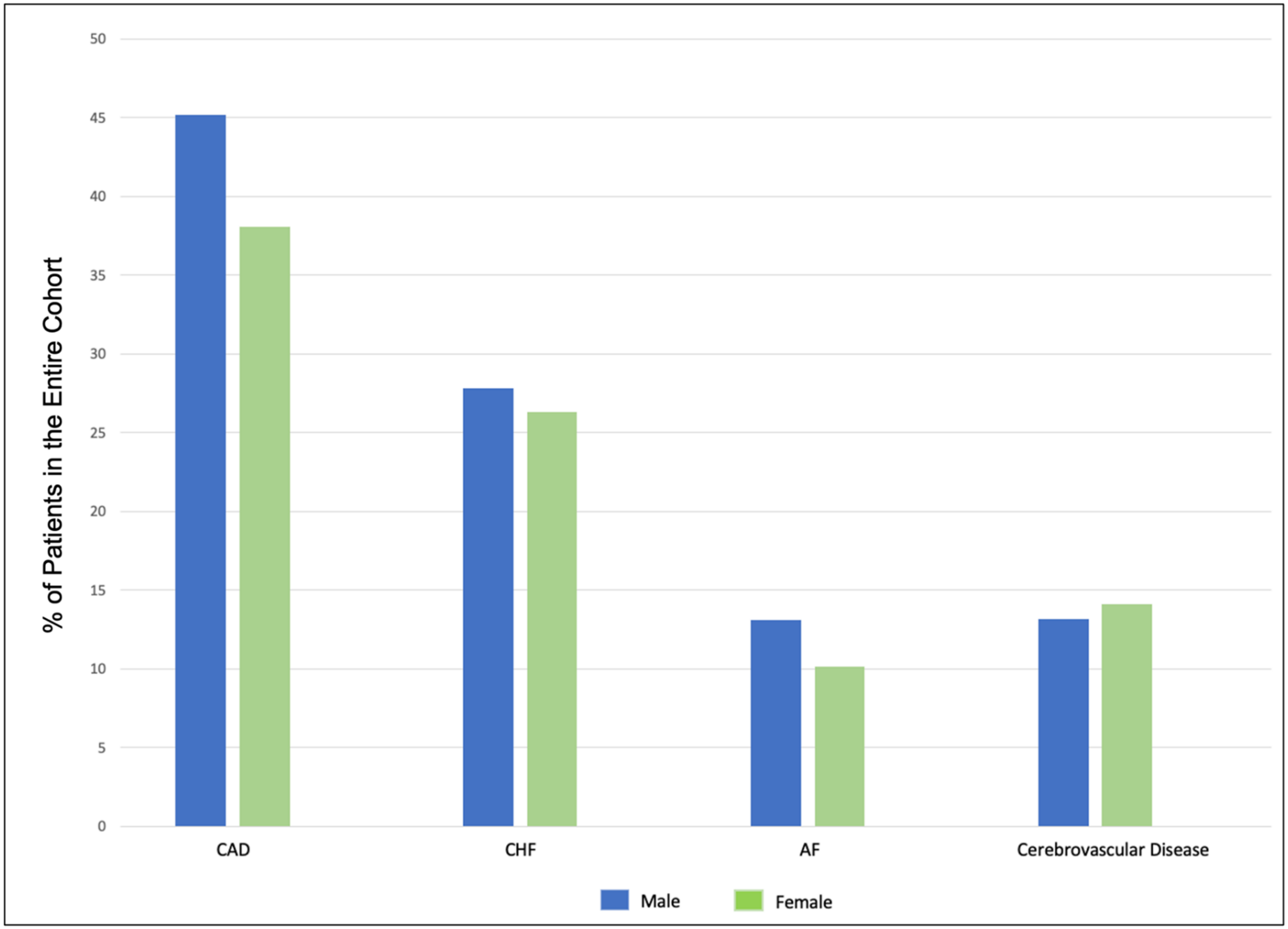

CAD-coronary artery disease; CHF-congestive heart failure; AF-atrial fibrillation or flutter.

© 2023 Eberly LA et al. *JAMA Network Open.*

B. Rates of Overall Cardiovascular Disease Risk Factors by Gender for the Entire Cohort, 2015-2019

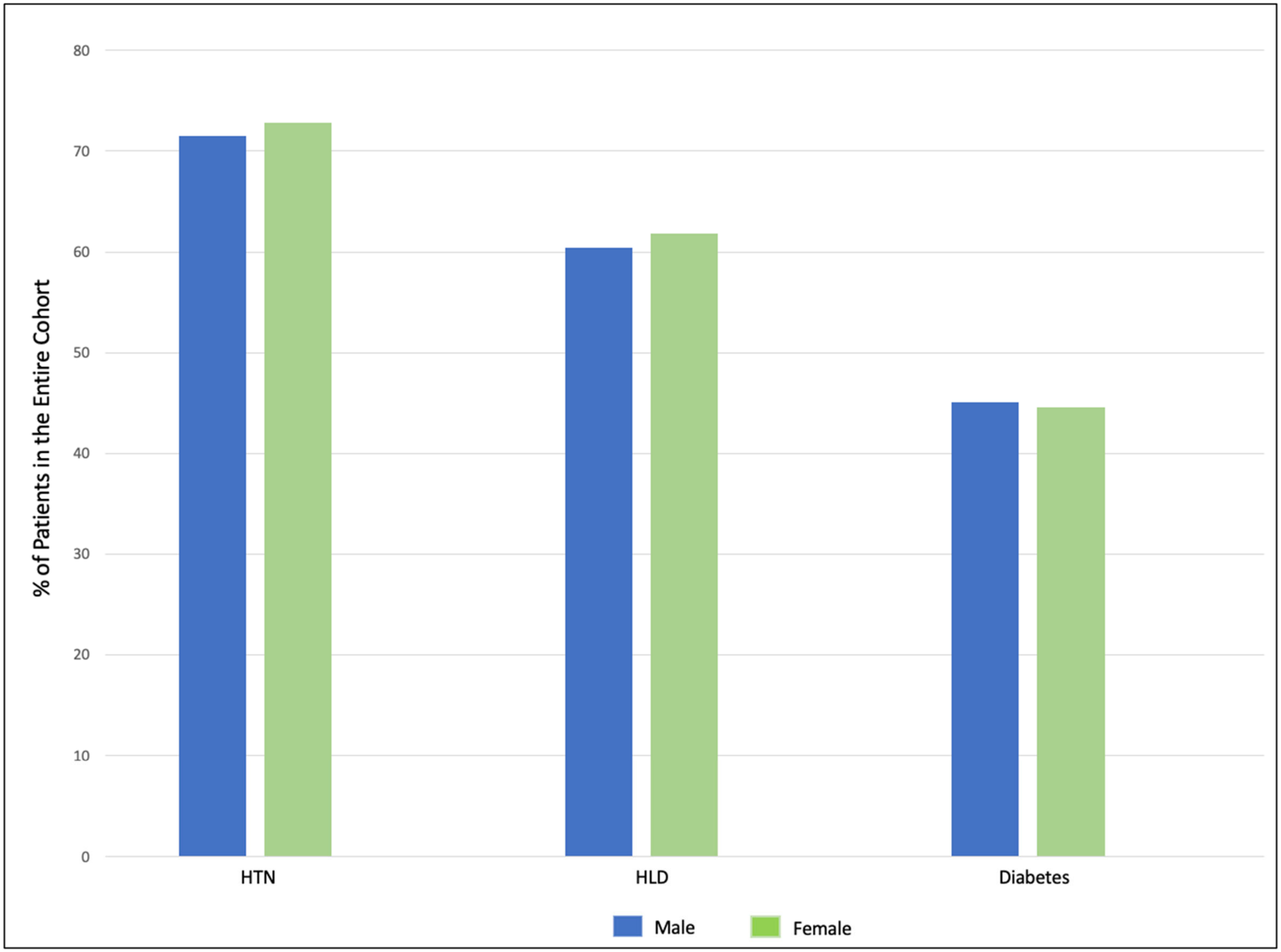

HTN-hypertension; HLD-hyperlipidemia.

© 2023 Eberly LA et al. *JAMA Network Open.*

eFigure 2. Rates of Overall Cardiovascular Disease Risk Factors by Gender for Patients without Established Cardiovascular Disease, 2015-2019

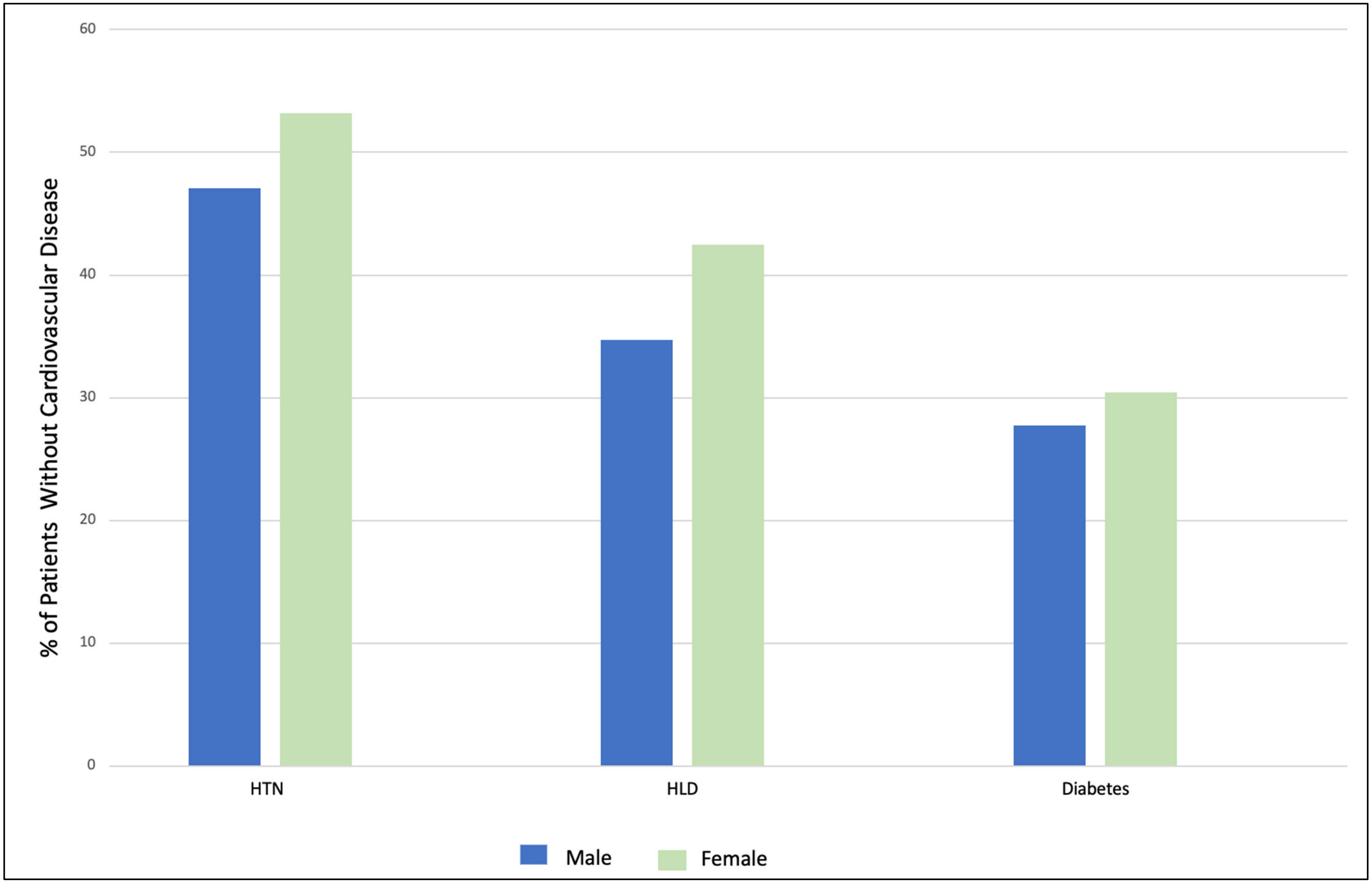

HTN-hypertension; HLD-hyperlipidemia.

© 2023 Eberly LA et al. *JAMA Network Open.*
