# Supplementary material for: Cardiovascular Disease Burden and Outcomes Among American Indian and Alaska Native Medicare Beneficiaries
Source: JAMA Netw Open. 2023 Sep 22;6(9):e2334923. doi: 10.1001/jamanetworkopen.2023.34923 (PMC10517375; doi:10.1001/jamanetworkopen.2023.34923)
Supplement: Supplement 2. — Data Sharing Statement [file jamanetwopen-e2334923-s002.pdf]

Data Sharing Statement

Eberly. Cardiovascular Disease Burden and Outcomes Among American Indian and Alaska Native Medicare Beneficiaries. *JAMA Netw Open*. Published September 21, 2023. doi:10.1001/jamanetworkopen.2023.34923

Data
Data available: No

Additional Information
Explanation for why data not available: Data was purchased through a licensing agreement with CMS. Thus, we are unable to share raw data. We can, however, share all summary data tables.
